# G-1639A but Not C1173T* VKORC1* Gene Polymorphism Is Related to Ischemic Stroke and Its Various Risk Factors in Ukrainian Population

**DOI:** 10.1155/2016/1298198

**Published:** 2016-09-15

**Authors:** Yevhen I. Dubovyk, Viktoriia Yu. Harbuzova, Alexander V. Ataman

**Affiliations:** ^1^Department of Physiology, Pathophysiology and Medical Biology, Sumy State University, Sumy 40007, Ukraine; ^2^Scientific Laboratory of Molecular Genetic Research, Sumy State University, Sumy 40007, Ukraine

## Abstract

Vitamin K epoxide reductase complex subunit 1 (VKORC1) is integral 163-amino acid long transmembrane protein which mediates recycling of vitamin K 2,3-epoxide to vitamin K hydroquinone and it is necessary for activation of vitamin K-dependent proteins (VKDPs). Herein, the association between G-1639A (rs9923231) and C1173T (rs9934438) single-nucleotide polymorphisms (SNPs) of the* VKORC1* gene and ischemic stroke (IS) was tested in Ukrainian population. Genotyping was performed in 170 IS patients and 124 control subjects (total 294 DNA samples) using PCR-RFLP (polymerase chain reaction with following restriction fragment length polymorphism analysis) method. Our data showed that G-1639A but not C1173T polymorphism was related to IS, regardless of adjustment for age, sex, body mass index, smoking status, and arterial hypertension. The risk for IS in -1639A allele carriers (OR = 2.138, *P* = 0.015) was higher than in individuals with G/G genotype. Haplotype analysis demonstrated that -1639G/1173T and -1639A/1173C were related to increased risk for IS (OR = 3.813, *P* = 0.010, and OR = 2.189, *P* = 0.011, resp.), while -1639G/1173C was a protective factor for IS (OR = 0.548, *P* < 0.001). Obtained results suggested that -1639A allele can be a possible genetic risk factor for IS in Ukrainian population.

## 1. Introduction 

A large number of proteins require posttranslational modification for further activation. The one way of such modification is a change of multiple glutamic acid residues to *γ*-carboxyglutamate in the peptide sequences of proteins (*γ*-carboxylation). The biochemical system which is responsible for carrying out this modification is called the vitamin K cycle [1]. The functioning of this cycle results in the oxidation of vitamin K hydroquinone to vitamin K 2,3-epoxide and it is impossible without recycling of vitamin K 2,3-epoxide to vitamin K hydroquinone. The enzyme which mediates recycling of vitamin K 2,3-epoxide to vitamin K hydroquinone is called vitamin K epoxide reductase complex subunit 1 (VKORC1).

VKORC1 is integral 163-amino acid long transmembrane protein (18 kDa) which is widely expressed in many organs and tissues of the human and animal organisms (liver, salivary gland, prostate, lung, kidney, brain, bone, skeletal muscle, heart, etc.) [2]. It is necessary for activation of vitamin K-dependent proteins (VKDPs), which undergo posttranslation modification in vitamin K cycle. It is known that VKDPs include a number of clotting factors involved in the coagulation cascade (factors II, VII, IX, and X), anticoagulants (proteins C, S, and Z), proteins involved in bone and soft-tissue mineralization (matrix Gla-protein (MGP), Gla-rich protein (GRP), and osteocalcin) [3, 4], protein involved in differentiation of vascular smooth muscle cells (VSMCs), and platelet activation (growth arrest-specific 6 (GAS6)) [5]. Consequently, it can be assumed that dysfunction of VKORC1 might cause activity reduction of VKDPs and thus might lead to thrombosis, calcification, and inflammation of the vascular wall, and so forth. Such changes are essential steps for development of atherosclerotic lesions of the brain arteries, which often lead to ischemic stroke.

Ischemic stroke (IS) is a multifactorial disease at which development is determined by environmental and genetic factors. Since the discovery of the* VKORC1* gene in 2004 [6, 7], numerous studies about the relation of various single-nucleotide polymorphisms (SNPs) of the* VKORC1* gene to development of cardiovascular and cerebrovascular diseases were conducted [8–12]. There are some studies where association of the* VKORC1* G-1639A, C1173T, and T2255C polymorphisms with IS in a Chinese population has been investigated [13–15], but the data obtained in other ethnic groups remain controversial [16–20]. Role studying of the* VKORC1* G-1639A and C1173T polymorphisms in development of IS in Ukrainian and other Slavic populations has not been conducted. Thus, we have performed a case-control study on representatives of Ukrainian population with the aim of investigating the possible association of the* VKORC1* G-1639A and C1173T SNPs with IS in individuals who had different risk factors of atherosclerosis.

## 2. Materials and Methods

### 2.1. Subjects

In present study we selected 170 unrelated Ukrainian patients (42.4% women and 57.6% men) from 40 to 85 years of age (mean age (±SD) 64.7 ± 9.5) who had IS and had been under medical surveillance and outpatient treatment in the 5th Sumy Clinical Hospital since 2009 to 2011. A final diagnosis of IS was established on the basis of clinical, computed tomography, and magnetic resonance imaging investigations. Each case of IS was assessed according to the TOAST (Trial of Org 10172 in Acute Stroke Treatment) criteria [21] on the basis of anamnestic data and peculiarities of the clinical disease circuit, as well as the data of ultrasonic Doppler sonography of the main head artery and electrocardiograms. The patients with IS of cardioembolic origin and undetermined etiology were excluded from the studied group. The clinical characteristics of patients with IS included arterial blood pressure (BP), body mass index (BMI), composition of blood plasma lipoproteins, and indices of blood coagulation. The total cholesterol, HDL-cholesterol, LDL-cholesterol, and triglyceride levels determination was done in 157 with IS. Thus, the analysis of VKORC1 polymorphisms influence on lipid metabolism in stroke patients was performed only between these 157 cases.

The control group included 124 individuals with the absence of ischemic stroke and other cerebrovascular pathologies, which was verified using amnestic data, ECG test, blood pressure measurement, and carrying out generally accepted neurologic researches. It is well known that warfarin and similar oral anticoagulants are inhibitors of VKORC1. To avoid distortion of results about association between* VKORC1* gene polymorphism and IS only individuals who have never taken anticoagulant therapy were included to the case and control groups.

The patients of both groups were divided into the pairs of subgroups defined by sex, BMI (BMI < 25 kg/m^2^ and ≥25 kg/m^2^), and BP (nonhypertensive or hypertensive: systolic BP > 140 mmHg, diastolic BP > 90 mmHg).

The study was complied with the Declaration of Helsinki and approved by the Ethic Committee of Medical Institute of Sumy State University. Written informed consent from all the subjects was obtained before enrollment.

### 2.2. Genotyping of SNPs

Genomic DNA was extracted from white cells using GeneJET Whole Blood Genomic DNA Purification Mini Kit (Thermo Fisher Scientific, USA) according to the manufacturer's protocol.* VKORC1* promoter G-1639A (rs9923231) and first intron C1173T (rs9934438) polymorphisms genotyping was performed using PCR-RFLP. We used primers synthesized by Metabion (Germany). The reaction mixture of 25 mL volume contained 50–100 ng of DNA, 1.5 mM magnesium sulfate, 200 mM of each dNTP, 5 *μ*L 5x PCR-buffer, 20 pM of each primer, and 0.5 U of Taq DNA polymerase (Thermo Fisher Scientific, USA). PCR was carried out in a thermocycler GeneAmp PCR System 2700 (Thermo Fisher Scientific, USA).

The sequence of nucleotides in specific primers for* VKORC1* promoter G-1639A SNP was as follows: forward – 5′-GCCAGCAGGAGAGGGAAATA-3′ and reverse – 5′-AGTTTGGACTACAGGTGCCT-3′. Thermocycling conditions consisted of 94°C for 5 min, followed by 33 cycles of 94°C for 50 s, 61°C for 45 s, and 72°C for 50 s with a final extension step of 72°C for 5 min. For restriction analysis 6 *μ*L of the amplification products was incubated at 37°C for 20 h with 5 U* Msp*I (*Hpa*II) (Thermo Fisher Scientific, USA). In the case of guanine at position -1639 of the* VKORC1* promoter amplified fragment, which consisted of 290, bps was cut by* Msp1* into two fragments of 168 and 122 bps. Guanine to adenine substitution resulted in the loss of* Msp1* restriction site and fragment of the promoter (290 bps) could not be cleaved.

The polymorphism of the* VKORC1* first intron (C1173T) was analyzed using the following primers: forward – 5′-AAGATGAAAAGCAGGGCCTAC-3′, reverse – 5′-CCGAGAAAGGTGATTTCCAA-3′. Thermocycling conditions consisted of 94°C for 5 min, followed by 33 cycles of 94°C for 50 s, 60°C for 50 s, and 72°C for 55 s with a final extension step of 72°C for 5 min. 6 *μ*L of the amplification products (195 bps) was incubated at 37°C for 18 h with 3 U* Sty*I (*Eco130*I) (Thermo Fisher Scientific, USA). The presence of cytosine at position 1173 of the gene prevented the restriction and in the case of substitution for thymine* Sty*I cleaved the amplified fragment into two fragments 125 and 70 bps in length.

The restriction fragments were separated by horizontal electrophoresis (electrical field strength 10 V/cm) in 1.5% agarose gel containing 10 mg/mL ethidium bromide. Visualization of DNA fragments after electrophoresis was performed using ultraviolet transillumination.

### 2.3. Statistical Analysis

Most statistical analyses were performed using Statistical Package for Social Science software (SPSS, version 17.0, Chicago, IL, USA). Continuous data are expressed as mean ± SD; categorical data are presented as number and percentage value. All continuous variables were normally distributed (Kolmogorov-Smirnov and Shapiro-Wilk tests); thus the comparison between the groups was performed using ANOVA or two-tailed Student's* t*-test. Bonferroni's correction was used for multiple comparisons. Each SNP was tested for deviation from Hardy-Weinberg equilibrium using the Online Encyclopedia for Genetic Epidemiology Studies (http://www.oege.org/software/hardy-weinberg.html). The *χ* ^2^ test was used to compare genotype and haplotype distributions of* VKORC1* SNPs between case and control groups. To estimate the risk we calculated the odds ratio (OR) and 95% confidence interval (CI) for the four models of inheritance: dominant (major homozygous genotype as a reference), recessive (genotypes with major allele as a reference), overdominant (major and minor homozygous genotypes as a reference), and additive (heterozygous genotype and minor homozygous genotype with major homozygous genotype as a reference). The Akaike information criterion (AIC) was used for selecting the most probable inheritance model. Such risk factors for IS like age, sex, BMI, smoking status, and arterial hypertension were incorporated as covariates by multivariable logistic regression analysis. Linkage disequilibrium (LD) and haplotype frequencies were analyzed by Arlequin (version 3.1, University of Berne, Bern, Switzerland). All statistical tests were two-sided; *P* < 0.05 was considered significant.

## 3. Results

The clinical characteristics of 170 cases and 124 controls are shown in Table 1. No significant differences between the groups with respect to gender, smoking status, and BMI were noted (*P* = 0.294, 0.403, and 0.279, resp.), but the average age of the control group (76.7 ± 10.2) was considerably higher than in the case group (*P* < 0.001). The average level of fasting glucose and the average meaning of systolic and diastolic BP were higher in IS group than in controls (*P* < 0.001).

The genotype distributions of the two SNPs (G-1639A and C1173T) in controls (minor allele frequency (MAF) = 0.371 and MAF = 0.327, resp.) and cases (MAF = 0.476 and MAF = 0.412, resp.) were consistent with the Hardy-Weinberg equilibrium (*P* > 0.05).

The results of* VKORC1* G-1639A genotyping are shown in Table 2. The difference in the distribution of three genotypes (G/G, G/A, and A/A) between the cases and controls was significant (*P* = 0.027). When analyzed in women and men independently, the significant difference in genotype distribution was not revealed (*P* = 0.228 and *P* = 0.119, resp.). Division of IS patients into subgroups according to the presence or absence of known atherosclerosis risk factors allowed carrying out a comparative analysis of their genotype frequencies. Statistically significant differences for groups with overweight (BMI ≥ 25 kg/m^2^) and arterial hypertension were established (*P* = 0.025 and *P* = 0.003, resp.).

Analysis of G-1639A genotypic association with IS under the four common models of inheritance is presented in Table 3. Significant association was established in total group before and after adjusting for age, gender, smoking status, BMI, and arterial hypertension under dominant (*P*_obs_ = 0.009, *P*_adj_ = 0.015) and additive model (*P*_obs_ = 0.032, *P*_adj_ = 0.041 for G/A genotype, and *P*_obs_ = 0.017, *P*_adj_ = 0.028 for A/A genotype). Relative risk analysis estimated an increased risk for IS in minor allele carriers (OR_adj_ = 2.138, 95% CI = 1.157–3.953) and separately in patients with G/A (OR_adj_ = 1.979, 95% CI = 1.029–3.805) and A/A (OR_adj_ = 2.621, 95% CI = 1.110–6.191) genotypes compared for individuals with G/G genotype. Genotypic association of G-1639A was also revealed in women after adjustment for covariates of age, BMI, smoking status, and arterial hypertension under dominant (*P*_adj_ = 0.038, OR_adj_ = 2.848, 95% CI = 1.058–7.665) and additive (*P*_adj_ = 0.049, OR_adj_ = 2.888, 95% CI = 1.006–8.293) model. In men significant difference was present only in the crude additive model (*P*_obs_ = 0.049, OR_obs_ = 2.240, 95% CI = 1.002–5.007) but was lost after adjustment (*P*_adj_ = 0.077). The association between G-1639A SNP and IS was also found to be significant in individuals with BMI ≥ 25 kg/m^2^ under dominant model with or without the adjustment for gender, age, smoking, and arterial hypertension (*P*_obs_ = 0.008, *P*_adj_ = 0.016, OR_adj_ = 2.391, 95% CI = 1.180–4.843). Under observed additive model genotypic association was revealed for both G/A (*P*_obs_ = 0.029, OR_obs_ = 1.997, 95% CI = 1.072–3.723) and A/A (*P*_obs_ = 0.020, OR_obs_ = 2.478, 95% CI = 1.155–5.317) genotypes, but after adjusting for the risk factors the genotypic association remained for A/A genotype (*P*_adj_ = 0.021, OR_adj_ = 3.304, 95% CI = 1.199–9.106), and was lost for G/A genotype (*P*_adj_ = 0.054). The frequencies of G-1639A genotypes were different between the cases and controls with arterial hypertension either before or after adjustment for the covariates of gender, age, BMI, and smoking status under dominant (*P*_obs_ = 0.002, *P*_adj_ = 0.029, and OR_adj_ = 2.374, 95% CI = 1.091–5.166) and recessive (*P*_obs_ = 0.015, *P*_adj_ = 0.049, and OR_adj_ = 2.862, 95% CI = 1.003–8.169) model. Significant association under additive model after adjusting was revealed only for A/A genotype (*P*_adj_ = 0.029, OR_adj_ = 4.029, 95% CI = 1.153–14.077). No genotypic associations between* VKORC1* G-1639A polymorphism and IS in subjects with BMI < 25 kg/m^2^ and normal BP were revealed (*P* > 0.05). Dominant model had the lowest value of AIC in most subgroups (Table 3).

Table 4 indicates the results of* VKORC1* C1173T polymorphism case-control genotyping. The distribution of three genotypes (C/C, C/T, and T/T) between the cases and controls was similar (*P* = 0.178). Statistically significant differences in the C1173T genotypes distribution were also absent when subjects of comparison groups were divided by gender, BMI, and blood pressure.

Analysis of C1173T genotypic association with IS under the four common models of inheritance is summarized in Table 5. The link with IS was not found either in total group or subgroups by gender, BMI, and BP under different inheritance models. The association was absent both without and with adjustment (*P*_obs_ > 0.05, *P*_adj_ > 0.05). The lowest AIC value in most subgroups was observed for dominant model (Table 5).

Clinical characteristics of the subgroups stratified by* VKORC1* G-1639A genotypes in IS subjects are shown in Table 6. The statistically significant difference was revealed only for thrombin time (16.48 ± 3.2 s, 17.25 ± 4.1 s, and 15.26 ± 2.5 s, *P* = 0.013). Bonferroni's correction allowed establishing the significant difference between individuals with A/A and G/A genotypes (*P* = 0.010). In Table 7 clinical characteristics of IS patients according to* VKORC1* C1173T genotypes are presented. As shown, statistically significant differences for any comparison were not found.

The next step of the present study was the calculation of linkage disequilibrium (LD) between G-1639A/C1173T SNPs pair. Significant high LD (*D*′ = 0.809, *r* ^2^ = 0.518) was revealed. Therefore, estimation of haplotype frequencies was performed. Analysis of the G-1639A/C1173T haplotype distribution in case and control groups is presented in Table 8. Significant difference in frequencies of -1639G/1173T and -1639A/1173C haplotypes between IS subjects and controls was established; herewith individuals with these haplotypes had an increased risk for IS (*P* = 0.010, OR = 3.813, 95% CI = 1.268–11.298, and *P* = 0.011, OR = 2.189, 95% CI = 1.185–4.045, resp.). In contrast, -1639G/1173C haplotype frequency was significantly higher in the control group than in IS patients and it decreased the risk for ischemic stroke (*P* < 0.001, OR = 0.548, 95% CI = 0.393–0.765). Frequency of -1639A/1173T haplotype in both groups was similar (*P* = 0.218).

## 4. Discussion 

The data obtained in present work demonstrated that* VKORC1* G-1639A (A risk allele frequency 47.6%) but not C1173T (T risk allele frequency 41.2%) polymorphism was associated with IS in Ukrainian population. It has been shown that the risk for IS in patients with A/A and G/A genotypes (G-1639A polymorphism) was higher than for individuals with G/G genotype. It should also be noted that the risk for IS was increased when combining A/A or G/A genotype with hypertension or overweight. Moreover, haplotype analysis revealed that individuals with -1639G/1173T and -1639A/1173C haplotypes had an enhanced risk for IS; conversely the -1639G/1173C haplotype was related to reduced risk for IS.

*VKORC1* C1173T polymorphic variant (rs9934438) is located in the first intron and leads to substitution of cytosine to thymine at 1173 position of the gene. Our results demonstrated high LD between G-1639A/C1173T SNPs pair, which was consistent with the data obtained in other studies [13, 14, 22]. The G-1639A (rs9923231) single-nucleotide polymorphism is located in the second nucleotide of the E-Box (CA/GGGTG) of* VKORC1* promoter region and leads to guanine/adenine conversion at position -1639 of the gene. Such -1639G>A substitution creates the E-box binding site (from CGGGTG to CAGGTG), which attracts repressive E-box binding proteins [23]. Therefore, this polymorphism leads to changes in the* VKORC1* promoter activity and causes reduction of* VKORC1* mRNA production and enzyme expression. According to this, few studies showed that* VKORC1* mRNA expression was higher in tissues of subjects with G-1639G/C1173C genotypes compared to subjects with G-1639A/C1173T and A-1639A/T1173T genotypes [23, 24].

It allows suggesting that inhibition of vitamin K recycling in -1639A and 1173T allele carriers may cause insufficient *γ*-carboxylation of protein C, protein S, protein Z, MGP, GRP, and osteocalcin. This would result in increased risk of clot formation, calcification of arteries, and atherosclerotic plaques contributing to the atherosclerosis and its complications development. According to this, Teichert et al. [8] showed that T-allele of the* VKORC1* C1173T polymorphism was associated with a significantly higher risk of aortic calcification in the Caucasian. Tavridou et al. [12] revealed association between* VKORC1* G-1639A SNP and maximum carotid intima-media thickness in type 2 diabetes mellitus, which was explained by the higher prevalence of calcification in individuals with -1639A allele.

Previously, we also investigated an association of* VKORC1* T2255C polymorphism with IS in the same Ukrainian population [25]. It was shown that carriers of C/C genotype had a significant higher risk for IS than individuals with T/T genotype. Our findings were consistent with results demonstrated by Du et al. [15], who reported that* VKORC1* G-1639A and T2255C were associated with susceptibility to cardiovascular and cerebrovascular diseases (CCVD) in Chinese population. Herewith, individuals with A and C allele had an increased risk for CCVD.

On the other hand, Zhang et al. [14] investigated the contribution of* VKORC1* G-1639A and C1173T SNPs to ischemic cerebrovascular disease (ICVD) in Chinese Han population and reported that subjects carrying the -1639G (G risk allele frequency 11.4%) or 1173C (C risk allele frequency 7.4%) allele might be at increased risk of ICVD. Furthermore, the 1639G-1173C haplotype was a risk factor for ICVD, and 1639A-1173T was a protective factor. The researchers suggested that -1639G allele, which can increase* VKORC1* mRNA production, was associated with low sensitivity to vitamin K antagonists and higher risk of thrombosis. Similar result was obtained by Wang et al. [13], who identified natural haplotype block in* VKORC1* gene, which included five common noncoding SNPs (G-1639A, C1173T, C1542G, T2255C, and G3730A) with strong LD. Authors discovered that* VKORC1* 2255C allele, which can reflect G-C-G-C-A haplotype, increased almost twice the risk of stroke, coronary heart disease, and aortic dissection in Chinese population. In support of this, Shyu et al. [18] showed that -1639A allele (VKORC1 G-1639A polymorphism) had protective effect on the development of large-artery atherosclerotic stroke and was associated with reduced stroke risk in Taiwan population. It was explained that minor allele carriers may have lower concentrations of blood coagulation factors, leading to protection against vascular thrombosis and, consequently, to a reduced susceptibility for stroke.

At the same time, the most case-control studies in Europe and North America population did not reveal association between* VKORC1* SNPs and cerebrovascular diseases development. Thus, Ragia et al. [17] did not find significant difference in the* VKORC1* G-1639A (A risk allele frequency 42.6%) genotypes distribution among Greek Caucasian IS patients and matched controls. Moreover, minor -1639A allele was not associated with occurrence and clinical aspects of ischemic stroke. Authors proposed that lack of such association could be explained by combined effects of -1639A allele on vitamin K-dependent hemostatic and nonhemostatic proteins, affecting both clot formation and vascular calcification. Hindorff et al. [19] did not reveal significant association of five common* VKORC1* SNPs and haplotypes with myocardial infarction, ischemic stroke, and venous thrombosis on large scale study in North American population. In accordance with the above, two case-control studies carried out in Belgian and Southern German population by Lemmens et al. [20] and Arnold et al. [16], respectively, also did not show any association between* VKORC1* haplotypes and different subtypes of ischemic stroke.

Recently, a meta-analysis conducted by Li et al. [26] demonstrated that G-1639A and T2255C SNPs in* VKORC1* gene might contribute to the risk of cerebrovascular and cardiovascular diseases. Herewith, the above data showed that minor alleles in some populations can be the wild alleles in other populations, which leads to different interpretation of the results. It turns out that* VKORC1* genetic polymorphisms can lead to reduction of the thrombosis and atherosclerosis risk in some cases and increase the risk of clot formation and atherogenesis in other cases, contributing to development of cerebrovascular and cardiovascular diseases (as has been shown in our study). The mechanism of this duality is not fully clear. One explanation for this could be the widespread vitamin K deficiency among individuals of different populations [27]. In such case, the transport systems provide preferential targeting of phylloquinone to the liver to preserve coagulation, while less important Gla-proteins, which are synthesized in the extrahepatic tissues, do not receive vitamin K (according to the triage theory by McCann and Ames [28]). In this way, VKORC1 activity reduction due to genetic polymorphisms exacerbates the shortage of the vitamin K in the extrahepatic tissues and is compensated by the vitamin K in the liver.

Our case-control study has few limitations, which have to be taken into account during interpretation the results. First of all, study groups included quite low number of individuals and may not represent the general Ukrainian population. Small size sample is explained by the difficulty of the subject selection. Only individuals who have never been on oral anticoagulant therapy were included to case and control groups. Such people are quite rare but were necessary for our study (the most part of oral anticoagulants inhibits VKORC1 and may obscure the impact of* VKORC1* gene polymorphism on IS development). Second, it should also be noted that average age of the control group was significantly higher than in the stroke patients. It was a condition of the study design, because it allowed suggesting that control individuals had the presumably reduced risk for IS in the future. Additionally, both groups were similar in smoking and body mass index, but the arterial hypertension prevalence was higher among IS patients. This fact makes it impossible to draw firm conclusion about the impact of this risk factor on IS development in individuals with different* VKORC1* genotypes. Finally, more people should be enrolled in the study even as other* VKORC1* SNPs should be analyzed in order to make a definitive conclusion about* VKORC1* association with ischemic stroke in Ukrainian population. It will be the focus of our further research.

## 5. Conclusion 

In summary, this is the first report investigating the association between* VKORC1* G-1639A and C1173T polymorphisms and IS in Ukrainian population. Obtained results revealed that G-1639A but not C1173T polymorphism was related to IS. The risk for IS in -1639A allele carriers was higher than in major allele homozygotes. Moreover, -1639G/1173T and -1639A/1173C haplotypes were risk factors for IS, and -1639G/1173C haplotype was a protective factor for IS. Subsequent studies with larger number of participants are required to confirm our present results.

## Figures and Tables

**Table 1:** Clinical characteristics of the study groups.

Parameter	IS group (*N* = 170)	Control group (*N* = 124)	*P*
Age, years	64.7 ± 9.5	76.7 ± 10.2	<0.001
Sex, male/female	72/98	45/79	0.294
Current smokers, *N* (%)	50 (29.4)	31 (25.0)	0.403
BMI, kg/m^2^	28.2 ± 4.3	27.6 ± 5.0	0.279
Systolic BP, mmHg	167 ± 29.2	152.6 ± 23.4	<0.001
Diastolic BP, mmHg	95.4 ± 15.6	86.3 ± 12.4	<0.001
Fasting glucose, mmol/L	5.92 ± 1.5	5.29 ± 0.7	<0.001

Categorical variables were compared by *χ* ^2^ test and continuous variables by *t*-test.

**Table 2:** Genotypes distribution of *VKORC1* G-1639A polymorphism in patients with IS and control subjects with different risk factors.

Group	*N*	Genotype			*P*
		G/G (%) (95% CI)	G/A (%) (95% CI)	A/A (%) (95% CI)	
Total					

IS	170	49 (28.8) (22.0–35.6)	79 (46.5) (40.0–54.0)	42 (24.7) (18.2–31.2)	0.027
Control	124	54 (43.6) (34.8–52.3)	49 (39.5) (30.9–48.1)	21 (16.9) (10.3–23.5)	

Gender					

Women					
IS	72	18 (25.0) (15.0–35.0)	39 (54.2) (42.7–65.7)	15 (20.8) (11.5–30.2)	0.228
Control	45	18 (40.0) (25.7–54.3)	20 (44.4) (29.9–59.0)	7 (15.6) (5.0–26.2)	

Men					
IS	98	31 (31.6) (22.4–40.8)	40 (40.8) (31.1–50.6)	27 (27.6) (18.7–36.4)	0.119
Control	79	36 (45.6) (34.6–56.6)	29 (36.7) (26.1–47.3)	14 (17.7) (9.3–26.1)	

BMI					

BMI < 25 kg/m^2^					
IS	41	10 (24.4) (11.3–37.5)	22 (53.7) (38.4–68.9)	9 (22.0) (9.3–34.6)	0.629
Control	38	13 (34.2) (19.1–49.3)	18 (47.4) (31.5–63.2)	7 (18.4) (6.1–30.8)	

BMI ≥ 25 kg/m^2^					
IS	129	39 (30.2) (22.3–38.2)	57 (44.2) (35.6–52.8)	33 (25.6) (18.1–33.1)	0.025
Control	85	41 (48.2) (37.6–58.9)	30 (35.3) (25.1–45.5)	14 (16.5) (8.6–24.4)	

Arterial blood pressure					

Nonhypertensive					
IS	42	14 (33.3) (19.1–47.6)	19 (45.2) (30.2–60.3)	9 (21.5) (9.0–33.8)	0.852
Control	48	17 (35.4) (21.9–49.0)	19 (39.6) (25.8–53.4)	12 (25.0) (12.8–37.3)	

Hypertensive					
IS	128	35 (27.3) (19.6–35.1)	60 (46.9) (38.2–55.5)	33 (25.8) (18.2–33.4)	0.003
Control	73	36 (49.3) (37.9–60.8)	29 (39.7) (28.5–51.0)	8 (11.0) (3.8–18.1)	

*N*: number of subjects in the subgroups.

*P*: the likelihood of differences between IS patients and control group by the *χ* ^2^-criterion.

95% CI: 95% confidence interval.

**Table 3:** Analysis of G-1639A genotypic association with IS under four common models of inheritance.

Model	*P*_obs_	OR_obs_ (95% CI)	*P*_adj_	OR_adj_ (95% CI)	AIC
Total					

Dominant	0.009	1.905 (1.172–3.097)	0.015	2.138 (1.157–3.953)	19.27
Recessive	0.111	1.609 (0.897–2.888)	0.142	1.780 (0.824–3.847)	23.46
Overdominant	0.235	1.329 (0.831–2.125)	0.226	1.434 (0.800–2.571)	24.66
Additive^a^	0.032	1.777 (1.050–3.006)	0.041	1.979 (1.029–3.805)	20.83
	0.017	2.204 (1.149–4.227)	0.028	2.621 (1.110–6.191)	

Gender					

Women					
Dominant	0.090	2.000 (0.899–4.452)	0.038	2.848 (1.058–7.665)	15.94
Recessive	0.479	1.429 (0.533–3.832)	0.520	1.498 (0.437–5.135)	18.31
Overdominant	0.307	1.477 (0.699–3.124)	0.133	2.046 (0.804–5.207)	17.78
Additive	0.122	1.950 (0.836–4.549)	0.049	2.888 (1.006–8.293)	17.91
	0.178	2.143 (0.706–6.501)	0.149	2.747 (0.697–10.826)	

Men					
Dominant	0.058	1.809 (0.979–3.344)	0.082	2.102 (0.911–4.582)	18.08
Recessive	0.126	1.766 (0.853–3.656)	0.196	1.958 (0.708–5.415)	19.27
Overdominant	0.578	1.189 (0.646–2.187)	0.521	1.292 (0.591–2.823)	21.37
Additive	0.173	1.602 (0.813–3.154)	0.166	1.872 (0.770–4.551)	19.40
	0.049	2.240 (1.002–5.007)	0.077	2.830 (0.894–8.959)	

BMI					

BMI < 25 kg/m^2^					
Dominant	0.339	1.612 (0.606–4.288)	0.519	1.521 (0.426–5.434)	14.98
Recessive	0.697	1.246 (0.413–3.758)	0.898	0.907 (0.203–4.062)	15.75
Overdominant	0.577	1.287 (0.531–3.116)	0.500	1.481 (0.474–4.625)	15.59
Additive	0.380	1.589 (0.565–4.465)	0.479	1.615 (0.428–6.086)	16.98
	0.434	1.671 (0.462–6.051)	0.801	1.252 (0.218–7.183)	

BMI ≥ 25 kg/m^2^					
Dominant	0.008	2.150 (1.219–3.793)	0.016	2.391 (1.180–4.843)	18.11
Recessive	0.118	1.743 (0.869–3.498)	0.090	2.230 (0.882–5.638)	22.61
Overdominant	0.196	1.451 (0.825–2.552)	0.282	1.457 (0.734–2.892)	23.47
Additive	0.029	1.997 (1.072–3.723)	0.054	2.107 (0.989–4.489)	19.81
	0.020	2.478 (1.155–5.317)	0.021	3.304 (1.199–9.106)	

Arterial blood pressure					

Nonhypertensive					
Dominant	0.836	1.097 (0.458–2.625)	0.228	1.966 (0.655–5.904)	15.77
Recessive	0.690	0.818 (0.306–2.191)	0.612	0.717 (0.199–2.588)	15.66
Overdominant	0.588	1.261 (0.545–2.918)	0.114	2.382 (0.811–6.997)	15.52
Additive	0.689	1.214 (0.469–3.143)	0.161	2.268 (0.722–7.123)	17.49
	0.870	0.911 (0.298–2.782)	0.725	0.778 (0.192–3.154)	

Hypertensive					
Dominant	0.002	2.585 (1.417–4.717)	0.029	2.374 (1.091–5.166)	19.72
Recessive	0.015	2.822 (1.255–6.501)	0.049	2.862 (1.003–8.169)	22.64
Overdominant	0.327	1.339 (0.747–2.399)	0.574	1.230 (0.598–2.528)	28.46
Additive	0.021	2.128 (1.119–4.046)	0.095	2.042 (0.844–4.720)	19.26
	0.002	4.243 (1.722–10.453)	0.029	4.029 (1.153–14.077)	

CI: confidence interval; AIC: Akaike information criterion; *P*_obs_: observed *P* value; OR_obs_: observed odds ratio; *P*_adj_: *P* value adjusted for covariates of age, gender, smoking status, body mass index, and hypertension in total group; for age, smoking status, body mass index, and hypertension in gender group; for age, gender, smoking status, and hypertension in BMI group; for age, gender, smoking status, and body mass index in hypertension group; OR_ads_: odds ratio adjusted for covariates.

^a^First row in additive model describes comparison of heterozygous genotype with major homozygous genotype and second row comparison of minor homozygous genotype with major homozygous genotype.

**Table 4:** Genotypes distribution of *VKORC1* C1173T polymorphism in patients with IS and control subjects with different risk factors.

Group	*N*	Genotype			*P*
		C/C (%) (95% CI)	C/T (%) (95% CI)	T/T (%) (95% CI)	
Total					

IS	170	63 (37.1) (29.8–44.3)	74 (43.5) (36.1–51.0)	33 (19.4) (13.5–25.4)	0.178
Control	124	59 (47.6) (38.8–56.4)	47 (37.9) (29.4–46.4)	18 (14.5) (8.3–20.7)	

Gender					

Women					
IS	72	24 (33.3) (22.4–44.2)	32 (44.4) (33.0–56.0)	16 (22.2) (12.6–31.8)	0.154
Control	45	22 (48.9) (34.3–63.5)	18 (40.0) (25.7–54.3)	5 (11.1) (1.9–20.3)	

Men					
IS	98	39 (39.8) (30.1–49.5)	42 (42.9) (33.1–52.7)	17 (17.3) (9.9–24.9)	0.626
Control	79	37 (46.8) (35.8–57.8)	29 (36.7) (26.1–47.3)	13 (16.5) (8.3–24.6)	

BMI					

BMI < 25 kg/m^2^					
IS	41	16 (39.0) (24.1–54.0)	16 (39.0) (24.1–54.0)	9 (22.0) (9.3–34.6)	0.568
Control	38	19 (50.0) (34.1–65.9)	11 (28.9) (14.5–43.4)	8 (21.1) (8.1–34.0)	

BMI ≥ 25 kg/m^2^					
IS	129	47 (36.4) (28.1–44.8)	58 (45.0) (36.4–53.6)	24 (18.6) (11.9–25.3)	0.212
Control	85	40 (47.1) (36.5–57.7)	35 (41.2) (30.7–51.6)	10 (11.8) (4.9–18.6)	

Arterial blood pressure					

Nonhypertensive					
IS	42	16 (38.1) (23.4–52.8)	19 (45.2) (30.2–60.3)	7 (16.7) (5.4–27.9)	0.725
Control	48	22 (45.8) (31.7–59.9)	18 (37.5) (23.8–51.2)	8 (16.7) (6.1–27.2)	

Hypertensive					
IS	128	47 (36.7) (28.4–45.1)	55 (43.0) (34.4–51.5)	26 (20.3) (13.3–27.3)	0.140
Control	73	37 (50.7) (39.2–62.2)	26 (35.6) (24.6–46.6)	10 (13.7) (5.8–21.6)	

See Table 2.

**Table 5:** Analysis of C1173T genotypic association with IS under four common models of inheritance.

Model	*P*_obs_	OR_obs_ (95% CI)	*P*_adj_	OR_adj_ (95% CI)	AIC
Total					

Dominant	0.071	1.542 (0.963–2.467)	0.054	1.914 (0.989–3.449)	18.96
Recessive	0.275	1.418 (0.757–2.657)	0.701	1.165 (0.535–2.538)	21.00
Overdominant	0.333	1.263 (0.787–2.026)	0.055	1.811 (0.989–3.318)	21.28
Additive	0.135	1.475 (0.886–2.455)	0.078	2.058 (0.912–3.926)	20.76
	0.117	1.717 (0.874–3.373)	0.260	1.620 (0.700–3.749)	

Gender					

Women					
Dominant	0.096	1.913 (0.892–4.102)	0.109	2.115 (0.846–5.286)	16.83
Recessive	0.135	2.286 (0.774–6.751)	0.243	2.162 (0.593–7.879)	17.17
Overdominant	0.637	1.200 (0.563–2.556)	0.454	1.427 (0.563–3.617)	19.41
Additive	0.242	1.630 (0.720–3.690)	0.217	1.869 (0.692–5.046)	17.79
	0.069	2.933 (0.921–9.347)	0.131	2.886 (0.730–11.413)	

Men					
Dominant	0.347	1.333 (0.732–2.426)	0.165	1.744 (0.796–3.823)	17.38
Recessive	0.875	1.066 (0.483–2.352)	0.794	0.872 (0.311–2.444)	18.24
Overdominant	0.407	1.293 (0.704–2.375)	0.101	1.998 (0.874–4.569)	17.58
Additive	0.340	1.374 (0.715–2.640)	0.099	2.091 (0.871–5.018)	19.33
	0.619	1.241 (0.530–2.905)	0.756	1.192 (0.394–3.602)	

BMI					

BMI < 25 kg/m^2^					
Dominant	0.328	1.562 (0.639–3.818)	0.496	1.476 (0.481–4.527)	15.23
Recessive	0.923	1.055 (0.360–3.089)	0.536	0.637 (0.153–2.655)	16.18
Overdominant	0.347	1.571 (0.613–4.025)	0.208	2.213 (0.642–7.626)	15.30
Additive	0.292	1.727 (0.626–4.769)	0.260	2.112 (0.575–7.763)	17.06
	0.625	1.336 (0.418–4.268)	0.820	0.839 (0.184–3.830)	

BMI ≥ 25 kg/m^2^					
Dominant	0.122	1.551 (0.889–2.706)	0.053	2.142 (0.974–4.310)	18.40
Recessive	0.184	1.714 (0.774–3.796)	0.346	1.598 (0.603–4.236)	18.94
Overdominant	0.585	1.167 (0.671–2.031)	0.141	1.683 (0.842–3.367)	20.50
Additive	0.257	1.410 (0.778–2.556)	0.054	2.085 (0.989–4.397)	19.65
	0.099	2.043 (0.873–4.777)	0.118	2.322 (0.806–6.868)	

Arterial blood pressure					

Nonhypertensive					
Dominant	0.459	1.375 (0.592–3.194)	0.295	1.730 (0.620–4.828)	15.44
Recessive	1.000	1.000 (0.329–3.038)	0.628	0.701 (0.166–2.955)	15.99
Overdominant	0.457	1.377 (0.593–3.199)	0.157	2.167 (0.743–6.318)	15.43
Additive	0.423	1.451 (0.584–3.610)	0.181	2.157 (0.700–6.648)	17.34
	0.763	1.203 (0.362–4.001)	0.978	0.978 (0.209–4.584)	

Hypertensive					
Dominant	0.055	1.771 (0.989–3.173)	0.071	1.960 (0.944–4.066)	17.64
Recessive	0.242	1.606 (0.726–3.553)	0.362	1.571 (0.594–4.152)	19.93
Overdominant	0.307	1.362 (0.753–2.465)	0.246	1.558 (0.736–3.299)	20.31
Additive	0.115	1.665 (0.883–3.142)	0.116	1.901 (0.853–4.236)	19.42
	0.097	2.047 (0.877–4.775)	0.164	2.097 (0.740–5.946)	

See Table 3.

**Table 6:** Clinical characteristics of IS patients with different *VKORC1* G-1639A genotypes.

Parameter	G/G	G/A	A/A	Total	*P*
*N*	49	79	42	170	—
BMI	27.9 ± 3.9	28.6 ± 4.9	27.8 ± 3.6	28.2 ± 4.3	0.533
Systolic BP, mmHg	164.1 ± 32.5	169.7 ± 29.3	165.2 ± 24.8	166.9 ± 29.2	0.522
Diastolic BP, mmHg	94.9 ± 15.3	96.4 ± 15.4	93.9 ± 16.4	95.4 ± 15.6	0.692
Total cholesterol^a^, mmol/L	5.09 ± 1.5	5.02 ± 1.5	5.06 ± 1.6	5.05 ± 1.5	0.961
HDL-cholesterol^a^, mmol/L	1.07 ± 0.3	1.01 ± 0.3	0.99 ± 0.3	1.02 ± 0.3	0.452
LDL-cholesterol^a^, mmol/L	3.29 ± 1.4	3.24 ± 1.4	3.24 ± 1.5	3.26 ± 1.4	0.979
Triglyceride^a^, mmol/L	1.62 ± 0.7	1.69 ± 0.8	1.80 ± 0.9	1.70 ± 0.8	0.588
Prothrombin time, s	9.68 ± 1.9	9.51 ± 2.0	9.17 ± 2.2	9.48 ± 2.0	0.483
Thrombin time, s	16.48 ± 3.2	17.25 ± 4.1	15.26 ± 2.5	16.54 ± 3.6	0.013^b^
Fibrinogen, g/L	3.79 ± 1.3	3.89 ± 1.3	4.17 ± 1.1	3.93 ± 1.2	0.301
Fasting glucose, mmol/L	5.82 ± 1.4	6.11 ± 1.6	5.70 ± 1.5	5.92 ± 1.5	0.312

*N*: number of subjects; HDL: high density lipoprotein; LDL: low density lipoprotein.

^a^ *N* = 47 for G/G genotype, *N* = 70 for G/A genotype, and *N* = 40 for A/A genotype.

^b^Significant difference between A/A and G/A genotypes (*P* = 0.010) by Bonferroni's correction.

**Table 7:** Clinical characteristics of IS patients with different *VKORC1* C1173T genotypes.

Parameter	C/C	C/T	T/T	Total	*P*
*N*	63	74	33	170	—
BMI, kg/m^2^	28.2 ± 5.0	28.3 ± 3.8	28.0 ± 4.0	28.2 ± 4.3	0.961
Systolic BP, mmHg	166.6 ± 28.9	167.6 ± 32.3	166.4 ± 22.6	167.0 ± 29.2	0.973
Diastolic BP, mmHg	94.1 ± 13.9	96.6 ± 18.3	95.0 ± 11.7	95.4 ± 15.6	0.657
Total cholesterol^a^, mmol/L	5.09 ± 1.6	4.98 ± 1.4	5.13 ± 1.6	5.05 ± 1.5	0.862
HDL-cholesterol^a^, mmol/L	1.05 ± 0.3	1.01 ± 0.3	1.00 ± 0.3	1.02 ± 0.3	0.670
LDL-cholesterol^a^, mmol/L	3.25 ± 1.5	3.22 ± 1.4	3.35 ± 1.5	3.26 ± 1.4	0.910
Triglyceride^a^, mmol/L	1.74 ± 0.8	1.66 ± 0.8	1.72 ± 0.9	1.70 ± 0.8	0.820
Prothrombin time, s	9.56 ± 2.0	9.61 ± 2.1	8.99 ± 1.9	9.48 ± 2.0	0.313
Thrombin time, s	16.59 ± 3.5	16.73 ± 3.6	16.00 ± 3.6	16.54 ± 3.6	0.616
Fibrinogen, g/L	3.85 ± 1.2	3.85 ± 1.3	4.26 ± 1.1	3.93 ± 1.2	0.227
Fasting glucose, mmol/L	6.02 ± 1.6	5.85 ± 1.4	5.90 ± 1.7	5.92 ± 1.5	0.795

*N*: number of subjects; HDL: high density lipoprotein; LDL: low density lipoprotein.

^a^ *N* = 59 for C/C genotype, *N* = 69 for C/T genotype, and *N* = 29 for T/T genotype.

**Table 8:** Analysis of G-1639A/C1173T haplotype distribution in IS and control groups.

Haplotype	IS group		Control group		*P*	OR	95% CI
	2*N*	Frequency	2*N*	Frequency			
G-T	20	0.059	4	0.016	0.010	3.813	1.268–11.298
A-C	42	0.124	15	0.061	0.011	2.189	1.185–4.045
G-C	158	0.464	152	0.613	<0.001	0.548	0.393–0.765
A-T	120	0.353	77	0.310	0.281	1.211	0.854–1.717

*N*: number of subjects; OR: odds ratio; CI: confidence interval.
